# Factors affecting adoption, implementation fidelity, and sustainability of the Redesigned Community Health Fund in Tanzania: a mixed methods protocol for process evaluation in the Dodoma region

**DOI:** 10.3402/gha.v8.29648

**Published:** 2015-12-15

**Authors:** Albino Kalolo, Ralf Radermacher, Manfred Stoermer, Menoris Meshack, Manuela De Allegri

**Affiliations:** 1 Institute of Public Health, Medical Faculty, University of Heidelberg, Heidelberg, Germany; 2 Department of Community Health, St. Francis University College of Health and Allied Sciences, Ifakara, Tanzania; 3 Deutsche Gesellschaft für Internationale Zusammenarbeit, Lilongwe, Malawi; 4 Swiss Tropical and Public Health Institute, Basel, Switzerland; 5 Health Promotion and System Strengthening (HPSS) project, Dodoma, Tanzania

**Keywords:** micro health insurance, low enrollment, innovations to increase enrollment, Community Health Fund in Tanzania, process evaluation, mixed methods, complex interventions

## Abstract

**Background:**

Despite the implementation of various initiatives to address low enrollment in voluntary micro health insurance (MHI) schemes in sub-Saharan Africa, the problem of low enrollment remains unresolved. The lack of process evaluations of such interventions makes it difficult to ascertain whether their poor results are because of design failures or implementation weaknesses.

**Objective:**

In this paper, we describe a process evaluation protocol aimed at opening the ‘black box’ to evaluate the implementation processes of the Redesigned Community Health Fund (CHF) program in the Dodoma region of Tanzania.

**Design:**

The study employs a cross-sectional mixed methods design and is being carried out 3 years after the launch of the Redesigned CHF program. The study is grounded in a conceptual framework which rests on the Diffusion of Innovation Theory and the Implementation Fidelity Framework. The study utilizes a mixture of quantitative and qualitative data collection tools (questionnaires, focus group discussions, in-depth interviews, and document review), and aligns the evaluation to the Theory of Intervention developed by our team. Quantitative data will be used to measure program adoption, implementation fidelity, and their moderating factors. Qualitative data will be used to explore the responses of stakeholders to the intervention, contextual factors, and moderators of adoption, implementation fidelity, and sustainability.

**Discussion:**

This protocol describes a systematic process evaluation in relation to the implementation of a reformed MHI. We trust that the theoretical approaches and methodologies described in our protocol may be useful to inform the design of future process evaluations focused on the assessment of complex interventions, such as MHI schemes.

There is a growing body of evidence from resource-limited countries on the potential of micro health insurance (MHI) schemes to advance progress toward Universal Health Coverage, thanks to improved access to care and reduced out-of-pocket spending (1–9). The concept of MHI refers to a form of health insurance that targets the poor (10). These schemes implement risk pooling and sharing of resources at the community level and are characterized by voluntary membership and prepayment of health services (1, 11). Many MHI schemes, however, face a variety of operational challenges that jeopardize their sustainability and effectiveness (12–14). Low enrollment has been repeatedly identified as the persistent limitation to the effective development of MHI, specifically so in sub-Saharan Africa (SSA) (4, 11, 15–19).

Across SSA settings, there have been efforts to overcome the problem of low enrollment (20–23). However, these initiatives still lack a systematic evaluation of their effects. To our knowledge, many studies have analyzed the problem of low enrollment (12, 15, 16, 24, 25), but only two studies have assessed the effects of initiatives adopted to attract people to enroll in MHI schemes (22, 23). A study in Burkina Faso (22) analyzed the effect of communication campaigns on adoption of a community-based health insurance scheme. Another study in Benin (23) on MHI initiatives concluded on the urgent need for future research to focus on unraveling the implementation process to enhance understanding of what factors contribute to the success or failure of initiatives aimed at increasing enrollment.

The Community Health Fund (CHF) model in Tanzania represents a form of district-based MHI scheme. CHFs are voluntary prepayment schemes whereby households pay flat-rate contributions set by each district based on the community's ability to pay. Premiums range from US$3 to US$18 (26), and the district receives a matching grant from the central government per household enrolled in the scheme (27). CHF started operating in some districts of Tanzania after a pilot program in the Igunga district in 1996 (14, 28). The 2001 CHF-Act made the implementation of CHF schemes mandatory for all districts in the country and determined that the Council Health Service Board (CHSB) should manage the scheme (27).

Like other MHI schemes, CHFs have repeatedly been found to attain penetration rates which rarely exceed 10% (14, 17). Literature states that (14, 21, 25) low enrollment is determined by unwillingness and inability to pay premiums, poor quality of health services, mistrust in the scheme's management, inadequacy of benefit package, unfavorable CHF design to attract and sustain enrollments, and beneficiaries’ lack of knowledge on how insurance works. In addition, some researchers have attributed low enrollment to the voluntary nature of the scheme and have explored the feasibility of making CHF compulsory (29).

To address low enrollment in CHF, multiple interventions have been implemented across districts. These initiatives have received support from various stakeholders, ranging from international donors (such as the Swiss Agency for Development and Cooperation (SDC) and Deutsche Gesellschaft für Internationale Zusammenarbeit (GIZ) to nongovernmental organizations (such as Africare, Compassion International, and the International Centre for Development and Research (CIDR)), as well as various local cooperative unions (21, 30). The interventions reported in the literature include group enrollment initiatives (28), public–private partnerships (30), introduction of buffer stocks of medicines in health facilities (21), pro-poor funding strategies (21), and the inclusion of hospital care in the CHF package (21). Recently, among the many initiatives, the Redesigned CHF program has been introduced in the Dodoma region with the aim to strengthen CHF structures and increase enrollment in the scheme.

There has been a limited effect of the aforementioned initiatives on enrollment (17, 21, 31). Yet, as observed in relation to the situation in the continent as a whole, because of a lack of process evaluations accompanying the implementation of these reforms, it is difficult to ascertain whether the poor results are to be attributed to factors related to the design of the interventions or to weaknesses in implementation.

Literature attests to the importance of comprehensive evaluation studies that explicitly link implementation processes to program outcomes (32–34). This need arises because the level and process of implementation affect the programs’ outcomes (35). In addition, assessment of the implementation processes is essential for assessing internal and external validity of the intervention. Assessing the implementation process of complex interventions (36) helps to 1) provide feedback for improving the intervention, 2) replicate an intervention in other settings, 3) interpret the outcomes of the intervention, and 4) appraise the generalizability and the transferability of the intervention.

It follows that process evaluations offer potential to describe the mechanisms through which a given intervention produces certain outcomes, documenting both expected and unexpected effects. In particular, process evaluation focusing on a Fidelity of Implementation (FOI) approach, that is, evaluations focused on ascertaining whether a given program has been implemented as intended, provide additional explanations in relation to the intervention outcomes, as it has been demonstrated that FOI mediates outcomes (33, 34). FOI evaluations also help to avoid type-III errors, that is, falsely attributing the lack of effect of a given intervention to the intervention itself rather than to weaknesses in its implementation (33, 34, 37). Specific to the implementation of MHI schemes, process evaluation can be instrumental in explaining the complexities of MHI initiatives aimed at increasing enrollment and the context within which such innovations are implemented.

In this paper, we describe a process evaluation protocol aimed at evaluating the implementation of the Redesigned CHF in the Dodoma region of Tanzania. Our study follows the implementation of the scheme for its first 3 years. Specifically, our study examines the extent of adoption (including the stakeholders’ response to the intervention) and FOI as well as the factors that influence the two, in the light of the scheme sustainability.

## Methods

### Research settings

#### Study area

The study will be carried out in the Dodoma region of Tanzania. Dodoma is located in central Tanzania and covers 41,311 km^2^ with a population of 2,083,588 people (38). It is divided into seven districts: Dodoma Municipal, Bahi, Chamwino, Kondoa, Mpwapwa, Kongwa, and Chemba. The region's seven districts are subdivided into 28 divisions, 190 wards, and 543 registered villages. Less than 20% of all people reside in urban areas (38). The economy of Dodoma is based on subsistence agriculture and animal husbandry. The region experiences frequent food shortages because of its arid climate (39).

There is high demand for health care because of high disease burden, as elsewhere in Tanzania (40, 41). By 2013, the Dodoma region counted 360 health facilities, of which 81% are government owned, 6% private-for-profit, 10% faith based, and 3% belonging to parastatal organizations (42). Healthcare financing comes from central government grants, development partners’ support, and local revenues generated through out-of-pocket payments and insurance schemes. The CHF is the main voluntary prepayment scheme intended to cover the informal sector, whereas the National Health Insurance Fund (NHIF) is a mandatory prepayment scheme for civil servants. The uptake of CHF in Dodoma barely reaches 10% (17). The contribution of private (nonprofit and for-profit) health insurance schemes is minimal (43).

#### The Intervention

The setting for our study arises within a major intervention, ‘CHF Iliyoboreshwa’, translated and hereafter referred to as the Redesigned CHF, which has been implemented in the entire Dodoma region since July 2011. This intervention, funded by SDC through a bilateral agreement with the Tanzanian government and technically supported by the Swiss Tropical and Public Health Institute (Swiss TPH) and the Micro Insurance Academy (MIA), aims at enhancing enrollment by strengthening structures and creating new motivation for people to join the scheme. Vodacom Tanzania supports the Internet and communication services for the data management system.

The intervention builds on the results of a situational analysis of the former (standard) CHF program which revealed structural problems (17). The major weaknesses were 1) lack of separation between purchaser and provider roles, that is, the CHSB represents both the interests of healthcare providers and the interests of CHF members; 2) a weak data management system which resulted in the unavailability of data for monitoring purposes, re-enrollment, or claims of matching grants; 3) a weak enrollment strategy that relied on passive enrollment in health facilities; and 4) lack of incentives for health service providers in the scheme, that is, problems with reimbursing health facilities, the poor quality of health services, insufficient feedback mechanisms, and problems with member identification (17).

To address the problems listed above, the Health Promotion and System Strengthening (HPSS) project developed the Redesigned CHF program which includes: 1) a comprehensive re-organization of the CHF structures to clearly distinguish the purchaser (CHF) from the provider (healthcare facilities) function; 2) installation of an Insurance Management Information System (IMIS) to empower the CHF with a comprehensive data management system that includes membership enrollment through mobile phone technology, contribution management, claim processing and payment, as well as member feedback collection; 3) a close-to-client enrollment strategy, which relies on enrollment officers (EOs) recruited at the village level; 4) active community sensitization and mobilization campaigns on CHF; and 5) review of the CHF benefit package, expanding the range of services to include hospitalization and portability of cards within the region. To participate in the Redesigned CHF program, the seven districts of the Dodoma region signed an agreement with HPSS project and adopted the CHF Standard Operation Procedures (SOP) manual.

The Redesigned CHF operations are implemented at four levels and comprise the community members (level one), village implementation teams (level two), district implementation teams (level three), and regional-level actors (level four). Village implementers (Village executive officers (VEOs) and EOs) work directly with the community either to handle enrollments or to mobilize people to enroll in the scheme. The district council with its CHF board, CHF management team, and CHF officers has the full responsibility for implementing the scheme in the area of its jurisdiction. Technical backstopping to the districts is provided by regional-level actors through the HPSS project regional advisory board (RAB). Chaired by the regional administrative secretary, the RAB has members from central government, the regional secretariat, representatives of each of the districts, and other stakeholders with a stake in Redesigned CHF, such as SDC and NHIF. Political leaders at all levels are expected to motivate people to join the scheme.

### Conceptual framework

**Fig. 1:**
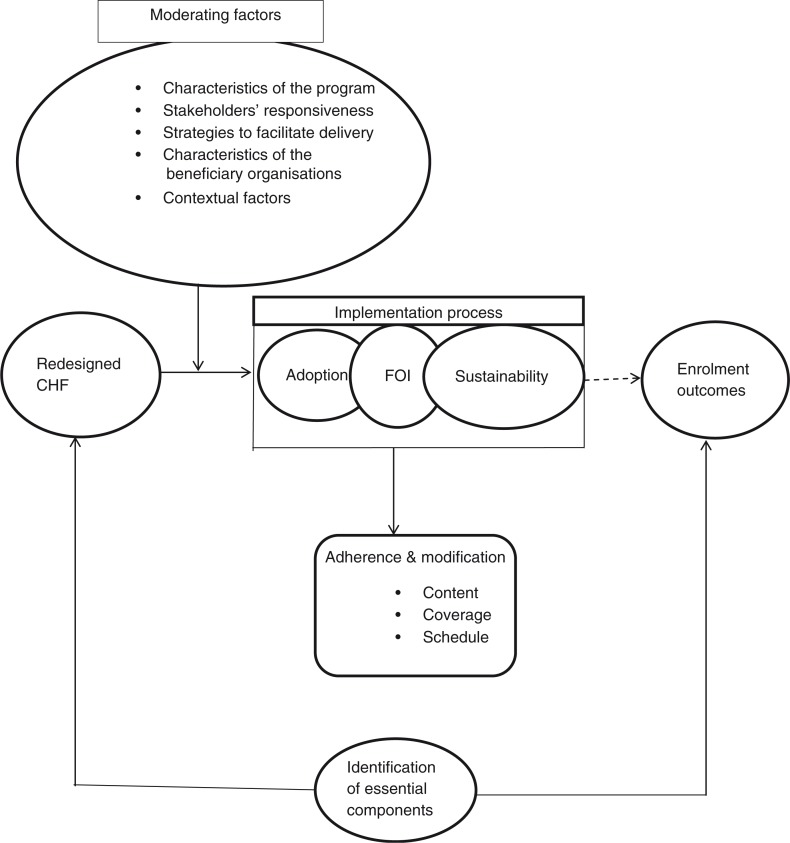
Process evaluation Conceptual framework (Concepts from Diffusion of Innovation theory (44) and Fidelity of Implementation Framework (33).

This study relies on a conceptual framework (Fig. 1) that is informed by the Diffusion of Innovations Theory (44) and by the FOI Framework (33).

The Diffusion of Innovations Theory addresses the complexity of getting innovations diffused into a community. It distinguishes four phases – dissemination, adoption, implementation, and sustainability – as crucial steps to the adequate diffusion of innovations (35, 44, 45). It recognizes that many theoretically effective innovations do not adequately reach the targeted communities because of issues that arise during any of these phases, particularly during the implementation phase (35). In line with Rogers’ proposition (44), making innovations diffuse successfully in communities originates from the way the innovation interacts with the human capital involved in the implementation process, as well as the context within which the innovative intervention is being implemented. Furthermore, the beneficiaries of the intervention need credible assurance that the changes introduced will not result into regrettable experiences, such as financial loss, humiliation, or waste of time.

The FOI Framework (33) provides in-depth understanding of the implementation processes of a given intervention. This framework considers adherence to the original program model and the factors that affect adherence as complementary parts of a comprehensive approach to measuring and understanding implementation.

In our conceptual framework, we use constructs from the two theories above to understand the implementation processes of the Redesigned CHF program. We combined the two theories as we aim to be comprehensive in identifying both the issues related to the adoption of the program and to its actual functioning as a health insurance scheme. This study defines the implementation process as a continuum from adoption through FOI to sustainability of the program processes.

In our study, we define adoption as the degree to which the intervention is integrated in the beneficiary organization structures and is practiced by them as the best course of action available to improve enrollment in CHF. In the course of understanding adoption, the study also attempts to understand whether there is rejection (active or passive) to the intervention. We define implementation as the way the Redesigned CHF program is delivered in the beneficiary districts.

We define FOI as the extent to which the program under observation (including its contents and processes) is implemented as designed (i.e. adherence to its original model amid the influence of moderating factors). Although other components may vary (be modified), past research recommends that core components be implemented in fidelity, as greater FOI of core components is associated with better program outcomes (35). We recognize that FOI acts as a potential moderator of the relationship between the innovation and its expected outcome (33, 46). Furthermore, we explore the process through which the intervention is delivered in relation to the expected and unexpected responses it elicits among the various stakeholders. We recognize that the implementation process will be influenced by the interaction between the new set of activities implemented in the region and all of the following moderating factors: program characteristics, stakeholders’ responsiveness (such as degree of involvement and motivation), strategies used to facilitate program delivery, organizational factors, and the context within which the intervention took place. In line with past research (33, 46), we recognize that the effects of these factors can be negative or positive and can only be determined when their influence is assessed systematically. Finally, we define sustainability as the extent to which a newly implemented innovation is maintained or institutionalized within a service setting and is running with stable operations (47, 48).

### Steps guiding the design and implementation of the process evaluation

To conduct this process evaluation, we rely on the following sequential steps: 1) identification of the essential intervention's components and the development of a comprehensive Theory of Intervention (TOI), 2) definition of the study design, 3) identification of a sampling strategy and data gathering procedures, 4) data analysis, 5) interpretation and integration of the results in the light of the conceptual framework, the TOI, and the study design.

#### Step 1: The identification of the essential components and the development of a theory of intervention

Developing a TOI represents the first essential step in the conduct of a process evaluation study (49). The TOI outlines the logical flow and the assumptions according to which a certain intervention is expected to lead to the desired outcomes (46, 50).

**Fig. 2:**
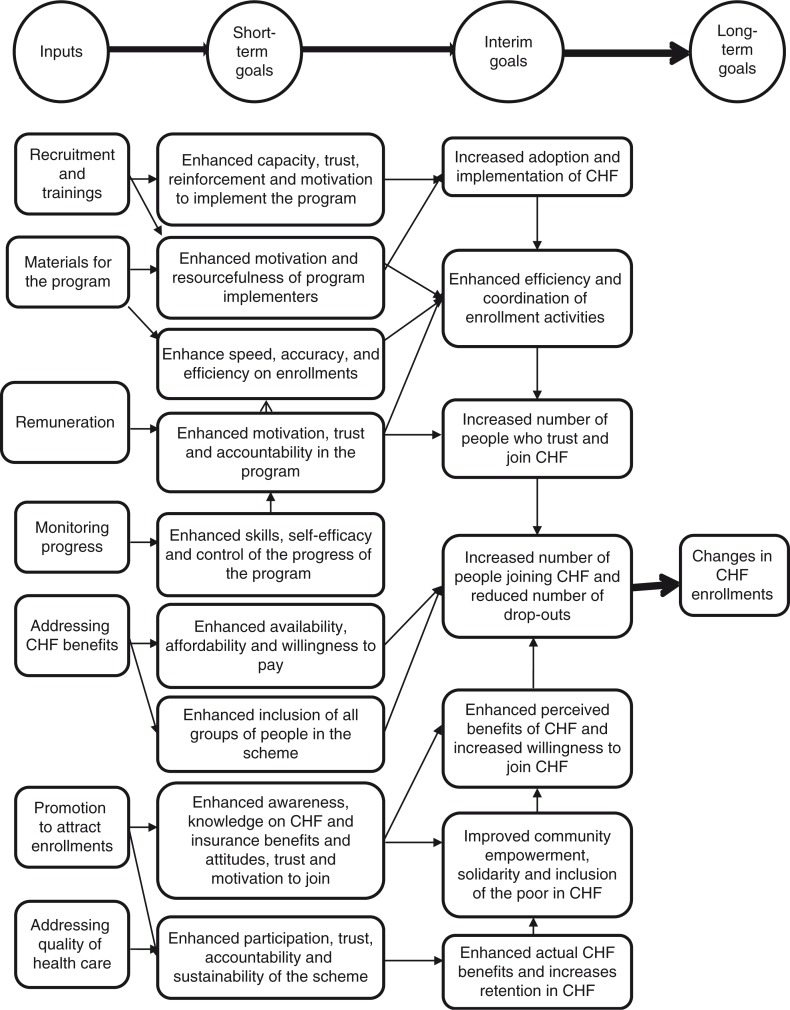
Theory of intervention (TOI) of the Redesigned CHF program.

Our TOI is outlined to show how the activities nested within the Redesigned CHF are expected to induce change in a series of intermediate outcomes, ultimately leading to an increase in enrollment rates in CHF. Figure 2 describes our TOI by providing a simplified visual representation of the intervention's ‘black box’ and by identifying essential activities critical to achieving the ultimate program outcomes. Given the difficulty of monitoring each and every activity within this complex intervention, we selected, in collaboration with the Redesigned CHF implementing team, a restricted series of 24 activities, judged as constituting the core of the intervention (Table 1). Furthermore, we grouped the 24 selected activities into seven functions to facilitate our analytical task (51). This allowed us to articulate the TOI with ease. The seven functions are: 1) recruitment and training, 2) materials for the program, 3) remuneration, 4) monitoring progress, 5) addressing CHF benefits, 6) promotion to attract enrollments, and 7) addressing the quality of health care. It needs to be noted explicitly that the TOI as it is currently presented represents an initial draft, based on an initial understanding of the intervention, and is therefore subject to modification as we acquire additional information through the conduct of the study itself.

**Table 1:** Essential components of the Redesigned CHF interventions

Essential component	Activities covered
Recruitment and training	Recruitment of key actors in CHF management structures
	Trainings and continuous coaching
Materials for the program	Provision of program materials
	Use of enrollment technologies (mobile phones, laptops, information data base)
Remuneration	Remuneration of all actors
Monitoring progress of the program	Meetings and workshops
	Supportive supervision and movement plan monitoring
	Monitoring of resource utilization
	CHF meetings at village level
Addressing CHF benefits	Review of premium
	Timely claims of matching grant from Ministry of Health and Social Welfare
	Benefit package development
	Improve quality of health services
	Pro-poor policies
Promotion to attract enrollments	Direct awareness campaigns
	Mass media campaigns
	Distribution of sales forces (IEC materials)
	Active enrollments at village level
	Community voice in stakeholders meeting
	Participation of community leaders in CHF advocacy
	Involvement of local initiatives (CSOs, traditional dance groups, etc.)
Addressing quality of health care	Availability of medicines and related supplies in health facilities
	Use of IMIS feedback tool
	Customer care to CHF clients

CHF, Community Health Fund; IMIS, Insurance Management Information System; IEC, Information, Education and Communication; CSO, Civil Society Organization.

#### Step 2: Definition of the study design

This study employs a cross-sectional, convergent parallel mixed methods design (52). The cross-sectional component refers to the one specific point in time of data collection and analysis, whereas the mixed methods component refers to the combination of quantitative and qualitative methods of data collection and analysis (52, 53). The convergent parallel component refers to the collection and analysis of the two independent strands of quantitative and qualitative data in a single phase. Results from the two strands are merged in order to look for convergence, divergence, contradictions, and relationships (52). We have applied a mixed methods approach because one single method would not be sufficient to capture the complexity of the implementation processes (49, 54, 55). This approach will allow us to explain the results in more detail, integrating multiple perspectives and identifying different plausible causal pathways between the activities observed and the expected outcomes.

The quantitative component measures the extent of program adoption, FOI, and sustainability of the intervention processes. The qualitative component is set to explore reasons behind adoption and FOI, the reactions of the stakeholders to the intervention and the contextual factors that affect the program's implementation.

#### Step 3: Identification of a sampling strategy and data-gathering procedures

Data will be collected from the four different levels of implementation and sources of information. Respondents include community members, village-level teams (EOs, VEOs, and healthcare workers), district-level actors (such as CHF management team members), and regional level actors (such as regional secretariat members and development partners).

The study will use several data collection tools for each level of data collection. In line with our mixed methods design, these include structured questionnaires, semi-structured open-ended interview guides to facilitate focus group discussions (FGDs) and in-depth interviews (IDIs), and the checklists for document review. Supplementary File outlines the single tools to be used in relation to the specific research questions and the evaluation domain (variables and their definitions) they refer to. To increase validity and reliability, we developed data collection tools following recommendation from literature (55), yet adjusted them to the local context. In addition, the tools will be piloted before actual data collection. The tools have been developed in English and then translated into *Kiswahili* (the widely spoken and official language in Tanzania). The different data collection tools are described in detail hereafter. The two strands of data will be collected concurrently by two different research teams in a parallel manner.

##### Structured questionnaires

Questionnaires will be administered to district and village implementation teams to capture their sociodemographic characteristics, knowledge on CHF, adoption of the Redesigned CHF, FOI of Redesigned CHF operations, and sustainability of the operations. We aim to capture respondents’ knowledge on the CHF scheme. We focus on their knowledge of CHF benefits, enrollment criteria, and differences between the old (standard) CHF and the Redesigned CHF.

To measure the way the teams adopt the Redesigned CHF, questions will focus on the functionality of Redesigned CHF structures and the usability of the guidelines in daily routines. FOI questions capture adherence to or departures from the CHF-SOP and the moderating factors. Sustainability questions focus on actions that are vital to sustain CHF operations. The questionnaire is composed of yes/no, Likert scale 1–5, and open-ended questions. We have developed two distinct questionnaires, to reflect the specific roles of the implementation teams being interviewed, that is, the district-level questionnaire and the village-level questionnaire.

Participants from the district level involve a census of implementers as per CHF-SOP manual, that is, from each of the districts, at least 10 members of the CHF implementation team. We use a multistage cluster sampling technique to select village-level participants. We include all of the geographic divisions of the seven districts (i.e. 28 divisions), as we aim to describe what happens across all parts of the concerned district. Each of the geographic divisions has an average of six wards and we will randomly select at least three wards per division and thereafter at least two villages from the selected wards. We divide the villages into two clusters based on the presence of a health facility, as we also wish to find out if there are differences in the implementation results between the two clusters. We will randomly select an equal number of villages from the clusters and include all the VEOs and EOs as study participants. We aim at selecting at least 20 villages from each of the districts to reach a sample size adequate for statistical analyses.

We use Cochran's formula for categorical data (56) to determine sample size for village-level participants and use FOI as a main implementation outcome variable given its expected direct link to program outcomes (33, 46). We assume adherence to core program components as being between 73 and 80% as reported by past research on complex intervention (46, 57) and allow for an estimated error margin of 5%.

###### Focus group discussions

FGDs will be conducted with community members (those enrolled and not enrolled in the CHF) in order to explore their perceptions and reactions to the program. Community members as ultimate beneficiaries of the scheme are included as participants because of their central role in the scheme. The group dynamics presented by FGDs allow in-depth understanding about a particular issue that cannot be obtained through personal interviews or a questionnaire. We will use semi-structured guides to collect information.

FGDs will be performed in only three districts selected purposively depending on percentage increase (speed) of CHF enrollment (i.e. highest, medium, and lowest), and relative to the baseline values for the 3 years of program implementation. From the selected districts, we will select villages from the entire geographic division by first selecting three wards depending on enrollment speed and thereafter two villages from each ward depending on the presence of a health facility and the distance from the ward headquarters. The aim is to record reactions from diverse groups of community members.

We plan to carry out FGDs until we reach redundancy and saturation (58, 59), but expect to begin by setting a target of 24 FGDs. We agreed on 24 FGDs given our wish to ensure sufficient geographic distribution (since that the program is active in an entire region) while allowing for pragmatic feasibility in data collection and analysis. The direct engagement of the first author in the data collection process will ensure that preliminary analysis is already initiated on the field and that data collection can be continued should redundancy and saturation not be reached once the initial set of 24 FGDs is completed. Each FGD will be constituted of a homogenous group, with men and women, the young and the elderly being interviewed in separate groups. Separating the groups helps to remove barriers that exist among group members as a result of social roles or cultural norms that could interfere with the freedom to express their views (60).

###### In-depth interviews

We plan to conduct in-depth qualitative interviews with a wide range of key informants drawn from various levels of Redesigned CHF implementation. The key informants who occupy specific functions (regional and district leaders or technical advisors) in Redesigned CHF implementation will participate in the study by virtue of their positions, whereas others will be selected based on their expected knowledge about the scheme.

We use IDIs as a tool to obtain detailed information from the participants. The interview questions aim at understanding the experience of implementing the scheme. We use semi-structured interview guides to collect information. We plan to carry out at least 12 interviews with regional level stakeholders and at least 36 interviews with district stakeholders. Participants from the districts will only come from three districts as in the FGDs.

###### Checklist for document review

We will use a checklist to collect information related to documentation of the implementation of the program. The checklist will be used to extract data from existing CHF documents and other permanent written products of the program.

The documents and permanent products set to be the source of data include program reports, day-to-day communications about the program as documented in the files (letters, memos, and meeting minutes), CHF policy documents, bylaws, strategic and operational plans, documentaries, and meeting minutes. In addition, the checklist will collect some secondary data from the IMIS-database.

The checklist will independently collect verifiable data to be triangulated with data from other tools. The checklist will be semi-quantitative (i.e. collecting both numerical and nonnumerical information) and will extract a mixture of data that is set to provide insights on implementation of the Redesigned CHF and its documentation.

#### Step 4: Data analysis

##### Analysis of quantitative data

Data from the questionnaires will be analyzed using standard statistical procedures. We quantify our dependent and independent variables because our interest is to respectively measure the extent of adoption, FOI, and sustainability, and find out what factors moderate adoption, FOI, and sustainability.

*Dependent variables*. Guided by our conceptual framework and parallel to the work of Proctor et al. (47) on types of outcomes in implementation research, we have defined adoption, FOI, and sustainability as implementation outcomes, and stakeholders’ satisfaction as a client outcome. The systematic assessment of service outcomes, such as enrollment outcomes and cost-effectiveness of the program, is beyond the scope of this study, but is considered for future research. We will, however, have access to routine program data measuring enrollment across the various districts. Thus, we will be able to link quantitative findings measuring implementation outcomes directly with enrollment to see how the former affect the latter. In addition, given the broad reach of our qualitative sample, we will also be able to explain some heterogeneity in enrollment across districts in the light of elements raised during the FGDs and the individual interviews.

Adoption will be measured in relation to three constructs: 1) adoption intensity, that is, the presence and functioning of the Redesigned CHF structures; 2) adoption rate, that is, the proportion of community members who join the scheme per year; and 3) degree of adoption, that is, the cumulative number of members enrolled in the Redesigned CHF.

FOI is measured as adherence to the program model as originally stipulated in the CHF-SOP and related program operational documents, such as training manuals, supervision guidelines, and movement plans. We will measure both structural and process fidelity. The former measures the way various structures have been institutionalized, while the latter measures the various processes related to the Redesigned CHF.

The proportion of procedures implemented as intended or modified is measured by the sum of scores of the responses to a set of questions representing a defined function as per TOI. We describe FOI per function and use it to calculate the overall FOI for a given implementation team. We will measure sustainability by a sum of scores of sustainability questions whereas sum scores of a set of satisfaction questions will measure stakeholders’ satisfaction.

*Independent variables*. The independent variables are the participants’ background variables and moderating factors. The background variables are age (years), sex (male/female), types of jobs (EOs, VEOs, etc.), duration of work (months), and level of education and knowledge on CHF. We measure knowledge on CHF as sum scores of correct responses to the knowledge questions.

We determine and measure the influence of moderating factors by the proportion they are mentioned by participants as moderators of implementation of the scheme. Included in our study as moderating factors are program characteristics, stakeholders’ responsiveness (such as degree of involvement and motivation), strategies used to facilitate program delivery, organizational factors, and contextual factors. They will be assessed by sum of scores of the responses to a set of questions representing a respective factor as detailed in Supplementary File.

##### Analysis of qualitative data

Analysis of qualitative data will be carried out by two independent researchers on the original transcripts and document-review extracts. Analysis will utilize the framework method (61) assisted by N-Vivo software (QRS-international). This method suits our study because of its flexibility (61) and offers a possibility of comparing results within and between levels at which data are collected (51). Qualitative analysis will proceed by reading interview transcripts and writing memos, coding the data, developing themes, and constructing a comprehensive narrative (52). Credibility will be established by triangulating data collection methods and sources and having at least two researchers independently code and analyze the transcripts (analyst triangulation) (52, 62).

#### Step 5: Integrating the quantitative and qualitative findings

Given the mixed methods nature of our study, we aim at integrating findings from across the data sets. Since in our case, quantitative and qualitative findings hold equal weight and are used to explore different aspects of the same phenomena, we aim at investigating where the findings converge, offer complimentary information, or appear to contradict each other. Integration here allows us to develop a composite, holistic, and cross-validated picture of the reality based on the results from both quantitative and qualitative data sets. Integration takes place after completion of data analysis and entails identifying similarities and differences, merging the results and discussing the meaning of the integrated results.

### Ethical consideration

The study will be conducted within a framework of the HPSS project in Tanzania (63). The study protocol was approved by the HPSS project and received ethical clearance from the National Institute for Medical Research (NIMR), Tanzania (Ref. NIMR/HQ/R.8a/Vol.IX/1821), and the Ethical Committee of the Medical Faculty of the University of Heidelberg, Germany (Ref.S-305/2014). Permission to conduct the study and consent to participate in the study will be sought from relevant authorities and participants, respectively. Participants will receive information about the purpose of the study and data protection.

### Protocol status

The protocol is under implementation. The entire timeline for completion of this study is 24 months and it is implemented in two stages. The theoretical stage (development of the conceptual framework, the TOI, and data collection tools) is estimated to take 9 months whereas the empirical stage (data gathering, analysis and dissemination of research findings) is estimated to take 15 months. Early field engagement began in July 2014 in order to develop and agree with implementing partners on a TOI, which could serve as the basis for the development of the protocol presented in this manuscript. Data collection was completed in early 2015. Data analysis will not have begun at the time of submission of this manuscript.

## Discussion

The lack of process evaluations of MHI interventions set to increase enrollments makes it difficult to identify and understand the contextual factors responsible for the success or failure of such initiatives. Our study aims to fill this knowledge gap by assessing the implementation of the Redesigned CHF. This is done with the dual objective of understanding the effect of the intervention and of shedding light on which elements contribute to the success or failure in implementing MHI interventions set to address low enrollments.

Our work situates itself within a context of fostering assessment of implementation just as much as the assessment of impacts of the interventions. This is important because process evaluation helps to determine the strength or weakness in implementation that could help to differentiate implementation failure and design failure when determining the impact of a given intervention (33, 35, 45, 47). In addition, process evaluations are essential for learning across settings, potentially contributing to reproducing interventions in other settings. Furthermore, our protocol intends to demonstrate the feasibility of conducting systematic process evaluations of complex interventions in the health systems of poor-resource countries.

We integrate multiple schools of thought and perspectives in methodological and analytical choices in order to conduct a robust assessment of the implementation of the scheme. Our approach of using mixed methods corroborates the work of Peters et al. (55), which describes it as a practical way to understand multiple perspectives, causal pathways, and outcomes of implementation research.

The study shall explore the challenges of implementing a complex intervention by looking at policy makers, frontline policy implementers, and consumer perspectives. While relevant from a conceptual point of view, this study will not explicitly explore the role of the funding agencies and the donor agencies in shaping the intervention content. The role of funding agencies and donors will be explored exclusively as a function of the environment, more specifically as a contextual factor influencing implementation processes. Looking at a scheme's implementation from multiple viewpoints helps to understand both the implementation processes and the reactions of the stakeholders, which are critical in explaining the implementation results (33, 47, 64). The fact that stakeholders do not receive the interventions passively, but interacting with them (49) points to the potentials of study results to inform decision making on scalable solutions to the intervention.

Successful implementation research requires good collaboration (48). Our research team is composed of intervention developers, implementers, and university-based researchers. This approach moves away from the old traditions of researchers being separated from implementers and enriches understanding of the intervention, its implementation in real-world settings, and methods needed for a trustworthy implementation study. In addition, it helps to bridge the research-implementation gap by bringing research findings closer to the implementers and policy makers (48).

Along with the strengths of our study protocol, we need to acknowledge some of its obvious limitations. In the first place, as mentioned above, we will not explicitly focus on exploring and understanding the role of the funding agencies in shaping the intervention content. It needs to be noted that our choice stems from the pragmatic need to narrow the focus to our process evaluation but may inevitably influence interpretation of its findings, since it may limit our ability to contextualize them in the light of all relevant elements. Second, the implementation of this study protocol may be influenced by factors such as field operational difficulties, cooperation from the implementing organization, and the researcher–participant relationship. There is the risk, albeit small, that these factors will influence what information we manage to successfully collect and thus, what interpretation and policy recommendations will follow from our research. Third, we must acknowledge the impossibility of conducting a prospective process evaluation, which relies on longitudinal data collection methods, given that our study team will gather data only a few years after the official launch of the Redesigned CHF. The impossibility of conducting longitudinal data collection motivated us to adopt a mixed method approach, relying on multiple data sources in order to recognize and incorporate multiple view points and to triangulate emerging interpretation at multiple levels. In addition, to strengthen our emerging interpretation and provide further contextualization of the findings, we will link the preliminary findings of the process evaluation with information on enrollment rates derived from the program. Unfortunately, however, we do not have access to information on the community health status, thus we cannot use this element to contextualize our findings. Fourth, our study is prone to recall bias as we intend to collect information concerning events that occurred in the past.

## Conclusions

In conclusion, the proposed research is intended to contribute to the understanding of the implementation processes of the Redesigned CHF program and helps to place the results of the intervention in context. In addition, evaluation studies like this help to differentiate implementation failure from design failure of a given intervention, which could not be identified by the impact of evaluation studies. The theoretical approaches and methodologies described in our protocol may be useful in informing the design of future process evaluations focused on the assessment of complex interventions.

## Supplementary Material

Factors affecting adoption, implementation fidelity, and sustainability of the Redesigned Community Health Fund in Tanzania: a mixed methods protocol for process evaluation in the Dodoma region
