# Supplementary material for: Factors affecting adoption, implementation fidelity, and sustainability of the Redesigned Community Health Fund in Tanzania: a mixed methods protocol for process evaluation in the Dodoma region
Source: Glob Health Action. 2015 Dec 15;8:10.3402/gha.v8.29648. doi: 10.3402/gha.v8.29648 (PMC4683988; doi:10.3402/gha.v8.29648)
Supplement: Factors affecting adoption, implementation fidelity, and sustainability of the Redesigned Community Health Fund in Tanzania: a mixed methods protocol for process evaluation in the Dodoma region [file GHA-8-29648-s001.pdf]

Supplementary file

Table 1: Evaluation plan of the Redesigned-CHF process evaluation

**Research question**	**Evaluation domain (variables)**	**Variable definition**	**Source of data**	**General process questions**	**Data collection tools**
1. What is the extent of adoption, implementation fidelity and sustainability of the redesigned CHF program?	Adoption	*Adoption rate*: The extent to which the intervention reaches the target population measured as a number of people enrolled in the CHF per year (by district/village) since the start of the redesigned CHF.	Program documents, Insurance management data base, Participants (*regional secretariat members, development partners, District leaders & CHF management team members*)	How is the intervention adopted by the District CHF structures? Why did the structures agree to participate?	Document review checklist, in-depth interview questionnaire
		Adoption intensity: The extent to which the program is actually integrated in the beneficiary organization structures and is actually practiced, measured by presence of key CHF actors and functional program structures			
		Degree of adoption: The population coverage of the intervention measured by a cumulative number of clients enrolled in the redesigned CHF scheme (by district) and socio-demographics since the start of the program			
	Fidelity of implementation	*Adherence:* The way the program model is implemented as intended, and will be a measure of FOI	Program documents, insurance management data base, participants (*regional secretariat members, development partners, District*	How much of the selected 24 series of intervention components implemented as planned? Were the intervention components implemented as often and for as long as planned? How and why do District CHF	Document review checklist, In-depth interview, questionnaire
		Content fidelity: Adherence to program model as stipulated in the CHF standard operating procedure manual (CHF-SOP) and related program documents with focus on essential components in the TOI			

**Research question**	**Evaluation domain (variables)**	**Variable definition**	**Source of data**	**General process questions**	**Data collection tools**
		Coverage fidelity: The coverage of the given procedures/activities measured as number of items (people, geographical areas and sessions) covered and the time used versus the time allocated.	*leaders & CHF management team members village executives, enrollment officers*)	structures achieve the pattern of reach they do? And what modifications/adaptations were introduced in the course of implementation? And why?	
		Modification: The extent to which the various CHF operations are modified to meet the conditions of the local settings, measured as a proportion of processes modified			
	Sustainability	*Funding allocation*: Amount of money allocated for CHF activities	(*regional secretariat members, development partners, District leaders & CHF management team members, village executives, enrollment officers*)	How and why are these processes sustained over time?	Document review checklist, in-depth interview, questionnaire
		Maintenance: Extent to which the program becomes routine and everyday culture and norms of the beneficiary organization including the degree to which the intervention processes are continued			
2. What factors influence adoption, implementation fidelity and sustainability of the intervention?	Program characteristics	*Comprehensiveness of program description*: The extent to which procedures or guidelines about the program are clear	Participants (*regional secretariat members, development partners, District leaders & CHF management team members*)	How specific and simple was the intervention description? What characteristics of the program made it easier to implement? How relevant was the program? Can results be seen more easily?	
		Relative advantage: The extent to which the program is perceived as advantageous over the current situation			
		Observability of result: The extent to which the positive results of the program are observable			

Research question	Evaluation domain (variables)	Variable definition	Source of data	General process questions	Data collection tools
	Stakeholder responsiveness	*Characteristics of implementers:* The various issues related to implementers' performance or motivation to implement the program components (availability of time, expectations, perceived support, level of knowledge and engagement in planning processes)	Participants (*regional secretariat members, development partners, District leaders & CHF management team members, village executives, enrollment officers, health care workers, members of the community*)	How do the implementers and target population respond?	Focus Group discussion, questionnaire, in-depth interview
		Satisfaction:* Implementers' subjective evaluation of the redesigned CHF program i.e., implementation strategy and materials and the implementation climate			
		Participation (involvement): The extent to which the various stakeholders are actually involved in the program and their reactions			
	Strategies to facilitate program delivery	*Positive implementation climate*: The extent the program is supported and rewarded within the organization assessed by presence of policies, guidelines, standard operating procedures and reward systems.	Participants (*regional secretariat members, development partners, District leaders & CHF management team members, village executives, enrollment officers*)	What strategies were used to facilitate implementation? And how did they influence the implementation processes?	In-depth interview questionnaire
		Technical support: Combination of resources offered to implementers to aid smooth implementation of the program, e.g. trainings, problem solving skills, mentorship etc.			
	Characteristics of beneficiary organization	*Leadership engagement*: The extent to which the (upper) leadership supports the program and their engagement in various program activities	Participants (*regional secretariat members,*	What organizational characteristics affected adoption and implementation of the	In-depth interview questionnaire

Research question	Evaluation domain (variables)	Variable definition	Source of data	General process questions	Data collection tools
		Policies and incentives: The extent to which the organizational policies support implementation of the intervention and the incentives in place to implement the program	*development partners, District leaders & CHF management team members, village executives, enrollment officers*)	program? What were the implementers' reactions to these strategies?	
		Resource availability: Level of resources made available for implementation of the program (financial, human and materials) and ongoing operations.			
	Contextual factors	*Socio-political context:* Politics and social policy environment influencing the intervention	Participants (*regional secretariat members, development partners, District leaders & CHF management team members, village executives, enrollment officers*)	Which is the wider context in which the intervention is being implemented? How does it influence implementation?	In-depth interview questionnaire
		Politico-economical context: Compatibility of the program with societal development and culture			

*Satisfaction acts both as a moderator of implementation but also as client outcome, i.e., one of the outcomes of the program is to satisfy clients (implementers and beneficiaries of the program).
